# An Uncommon Plant Growth Regulator, Diethyl Aminoethyl Hexanoate, Is Highly Effective in Tissue Cultures of the Important Medicinal Plant Purple Coneflower (*Echinacea purpurea* L.)

**DOI:** 10.1155/2013/540316

**Published:** 2013-12-23

**Authors:** Xiao-Lu Chen, Jun-Jie Zhang, Rong Chen, Qing-Ling Li, Yue-Sheng Yang, Hong Wu

**Affiliations:** ^1^College of Life Sciences, South China Agricultural University, Guangzhou 510642, China; ^2^Guangdong Key Laboratory for Innovative Development and Utilization of Forest Plant Germplasm, South China Agricultural University, Guangzhou 510642, China

## Abstract

We investigated the effects of various concentrations of diethyl aminoethyl hexanoate (DA-6) on the regeneration and growth of adventitious buds in *in vitro* purple coneflower cultures. Among the 3 types of explants tested, leaf explants required higher concentrations of DA-6 than petiole and root explants in order to obtain high regeneration rates, while root explants required the lowest concentration of DA-6. Additionally, explants with higher ploidy levels were more sensitive to the addition of DA-6, while explants with lower ploidy levels required higher concentrations of DA-6 to achieve its maximal regeneration rate. Interestingly, the application of a concentration that was conducive to the regeneration of explants with lower ploidy levels was inhibitory to the regeneration of explants with higher ploidy levels. Moreover, during the growth of regenerated buds, DA-6 application significantly improved plant height and weight, root weight, root thickness, root number, primary root length, total root length, and root/top ratio. Differences in the responses of explants to supplementation with DA-6 were also observed among explants with different ploidy levels, with buds having lower ploidy levels responding to lower concentrations of DA-6. Taken together, the results of the present experiments showed that proper application of DA-6 could increase *in vitro* culture efficiency in purple coneflower.

## 1. **Introduction**

Purple coneflower (*Echinacea purpurea *L.) is a popular medicinal plant used worldwide [1, 2], and various biotechnological attempts have been made [3] to improve characteristics of the plant by transforming genes [4], culturing anthers [5], and doubling the number of chromosomes [6]. Before all these goals can be realized, regeneration of adventitious buds from the cultures is a prerequisite step. Although there have been several reports on the regeneration of adventitious buds in purple coneflower [7–9], significant variations in the regenerative capacities of different genotypes have also been detected [10], suggesting the necessity for further improvement of *in vitro* regeneration culture techniques.

Researchers have successfully obtained haploid [5] and tetraploid [6] purple coneflower plants and subsequently compared the *in vitro *rooting abilities of these haploid, tetraploid, and parental diploid plants; their data demonstrated that buds with higher ploidy levels require greater concentrations of naphthaleneacetic acid (NAA) or indole-butyric acid (IBA) for the initiation of rooting [11]. Although these studies revealed high root induction rates, even for the tetraploid, survival rates after transplantation were generally low, indicating the poor quality of plantlets recovered by conventional methods.

Diethyl aminoethyl hexanoate (DA-6), a compound known to act as a plant growth regulator [12, 13], is routinely used in the field [14–17], but it is seldom used in tissue cultures [18, 19] and has not yet been tested in purple coneflower cultures. In this study, we investigated the sensitivity of specific tissues of purple coneflower plants with different ploidy levels to the addition of DA-6 in the medium. Our data suggested that the regeneration and growth of the regenerated buds could be effectively improved using DA-6.

## 2. Materials and Methods

### 2.1. Plant Materials

Original diploid purple coneflower plants were grown from seeds provided by the Company of Plantation Products (Norton, MA, USA). Tetraploid plants were obtained by *in vitro* treatment of diploid explants with colchicine, and plant regeneration was induced in these colchicine-treated explants [6]. Triploid plants were obtained by crossing diploid and tetraploid plants and growing the resulting seeds. Finally, hexaploid plants, similar to those of tetraploid [6], were obtained by treating the triploid explants with colchicine, and plant regeneration was then induced from the colchicine-treated triploid explants. The ploidy levels of these plants were confirmed by checking the chromosome numbers of root tip cells. Plantlets of diploid, tetraploid, triploid, and hexaploid genotypes were cloned by inducing axillary bud proliferation [20] from one individual plantlet, and the resulting plants were used in the following experiments.

### 2.2. Preparation of Explants

Leaves, petioles, and roots were isolated from *in vitro* maintained clones of intact diploid, triploid, tetraploid, and hexaploid plants, and explants were prepared by cutting these organs: leaf explants were cut to about 0.6 cm^2^, while petiole and root explants were cut to about 0.8 cm in length.

### 2.3. Preparation of Medium

Each culture bottle was filled with 40 mL medium and covered with a polycarbonate screw cap. Medium for inducing adventitious bud formation from explants was comprised of Murashige and Skoog (MS) basal elements, 3% sucrose, 0.3 mg/L IBA, and 0.01 mg/L NAA. Medium for culture of the regenerated buds was comprised of MS basal elements, 3% sucrose, 0.01 mg/L NAA, and various concentrations of DA-6. For addition to tissue culture, DA-6 was dissolved in water and added to the medium at the designated concentrations before autoclaving. All media were gelled with 0.45% agar and sterilized by autoclaving at 1.4 kg cm^−2^ for 20 min.

### 2.4. Maintenance of the Cultures

Cultures for regeneration and growth of adventitious buds were kept under cool-white light (about 40 *μ*mol m^−2^ s^−1^) with a 12 h photoperiod and a temperature range of 25–27°C.

### 2.5. Data Collection and Analysis

Data were collected 50 days after initiation of both the bud regeneration cultures and isolated bud growth cultures. In the bud regeneration cultures, the number of regenerated buds from each explant was counted; in the growth cultures of the isolated buds, weights of the plants and roots were recorded from fresh tissues, and the thickness of roots was measured under a microscope with an electronic scale. Statistical analysis of the data was carried out using SPSS 21.0 software, and significance differences among means were determined by Duncan's multiple range tests. Differences were considered significant when *P* values were less than 0.05.

## 3. **Results**

### 3.1. Effects of DA-6 on Bud Regeneration in Explants with Different Ploidy Levels

Leaf, petiole, and root explants were prepared from *in vitro* plantlets with different ploidy levels and were inoculated onto media supplemented with various concentrations of DA-6. Effects of DA-6 on the regeneration of adventitious buds were evaluated.

**Figure 1:**
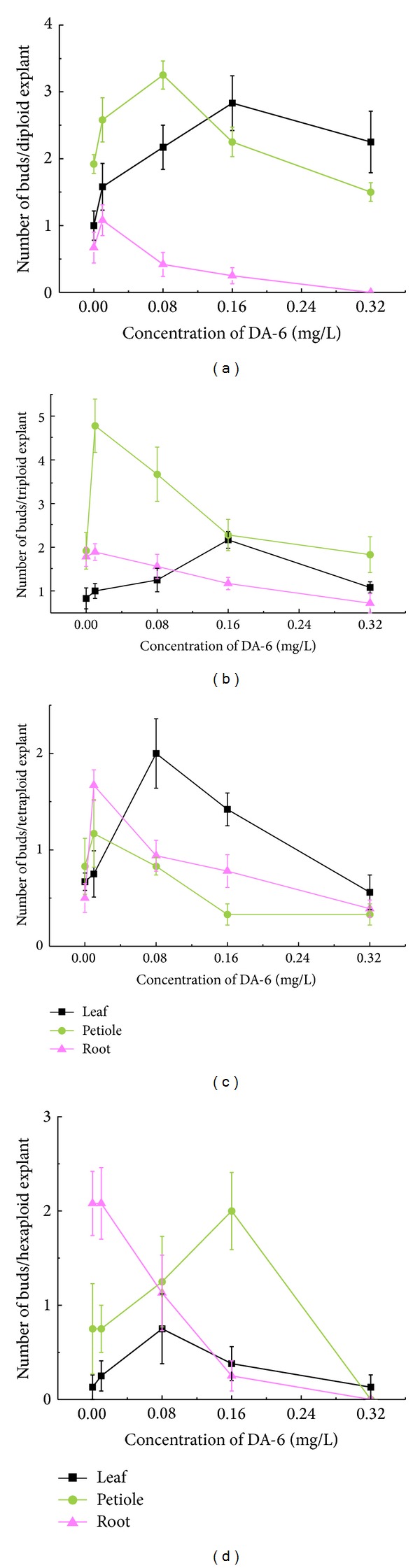
Effects of DA-6 on the regeneration of buds from different explants with different ploidy levels. (a)–(d) Diploid, triploid, tetraploid, and hexaploid, respectively.

**Figure 2:**
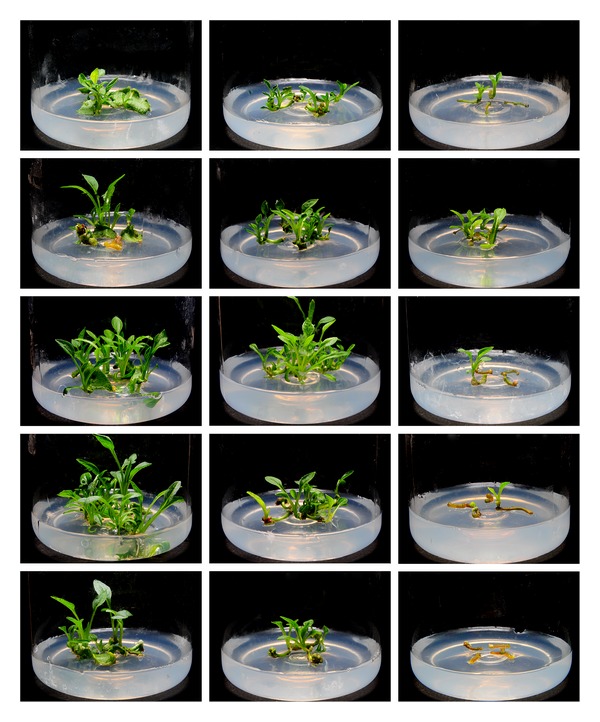
Comparison of the effects of DA-6 on regeneration of different diploid explants. Columns from left to right: explants of leaf, petiole, and root; rows from top to bottom: 0, 0.01, 0.08, 0.16, and 0.32 mg/L DA-6.

Our data, shown in Figures 1(a) and 2, demonstrated that DA-6 could stimulate the regeneration of adventitious buds in all types of diploid explants when used at suitable concentrations, which varied for each type of explants. Compared to petiole and root explants, leaf explants required the highest concentration of DA-6 (0.16 mg/L) in order to maximize the number of regenerated buds. Furthermore, leaf explants were not inhibited by bud regeneration at the highest concentration of DA-6 tested (0.32 mg/L); in contrast, this concentration decreased the number of regenerated buds by about 20% in cultures of petiole explants and completely suppressed regeneration in cultures of root explants.

**Figure 3:**
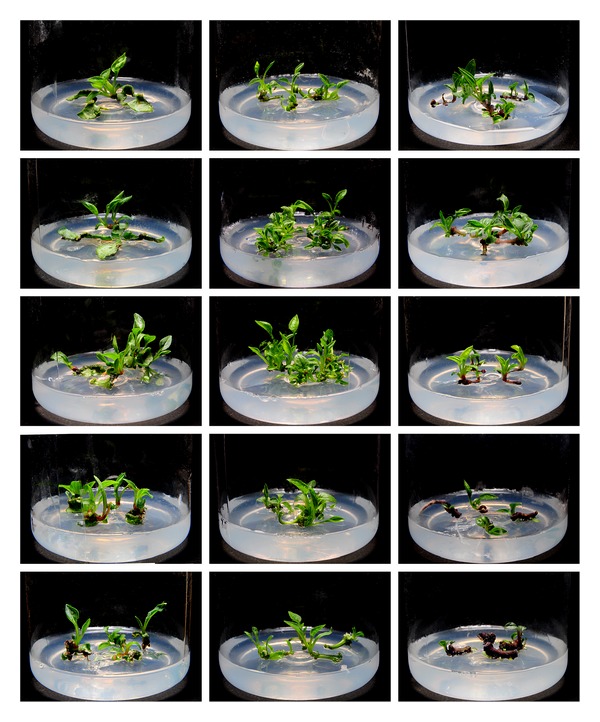
Comparison of the effects of DA-6 on regeneration of different triploid explants. Columns from left to right: explants of leaf, petiole, and root; rows from top to bottom: 0, 0.01, 0.08, 0.16, and 0.32 mg/L DA-6.

In triploid plants, DA-6 stimulated the regeneration of leaf explants at an optimal concentration of 0.16 mg/L (Figures 1(b) and 3). Compared to leaf explants, petiole explants required a much lower DA-6 concentration (0.01 mg/L) to achieve maximal regeneration. Although a small stimulatory effect could be observed when DA-6 was used at 0.01 mg/L for root explants, concentrations higher than 0.01 mg/L dramatically inhibited regeneration.

**Figure 4:**
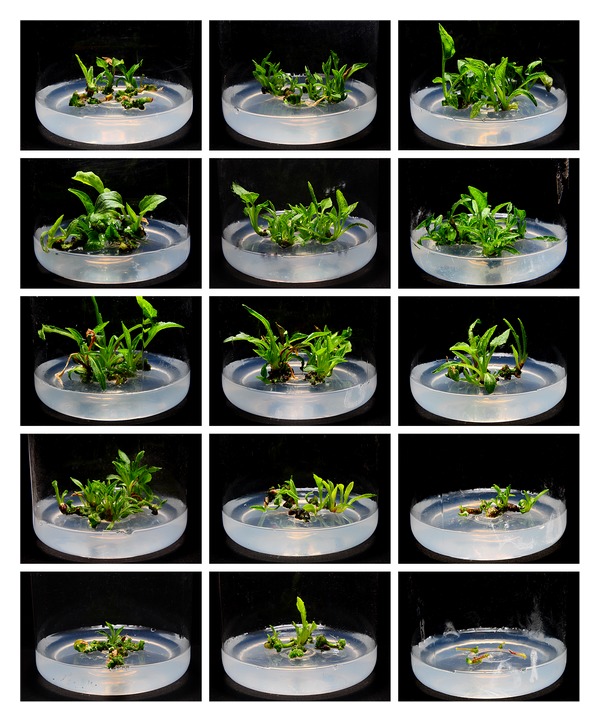
Comparison of the effects of DA-6 on regeneration of different tetraploid explants. Columns from left to right: explants of leaf, petiole, and root; rows from top to bottom: 0, 0.01, 0.08, 0.16, and 0.32 mg/L DA-6.

DA-6 also stimulated the regeneration of tetraploid leaf explants at an optimal concentration of 0.08 mg/L (Figures 1(c) and 4). Compared to leaf explants, petiole and root explants required a lower DA-6 concentration (0.01 mg/L) to achieve maximal regeneration. Concentrations of DA-6 as high as 0.32 mg/L were inhibitory to the regeneration of leaf and root explants, and 0.16 mg/L or higher DA-6 inhibited the regeneration of petiole explants.

**Figure 5:**
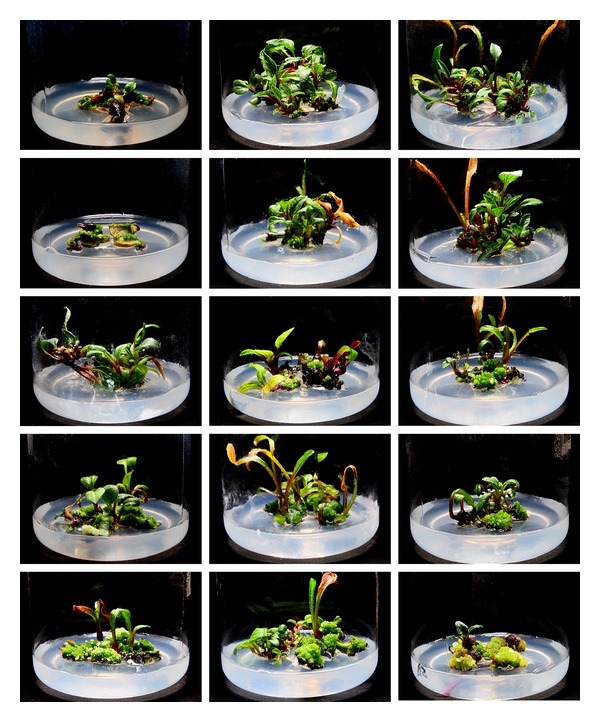
Comparison of the effects of DA-6 on regeneration of different hexaploid explants. Columns from left to right: explants of leaf, petiole, and root; rows from top to bottom: 0, 0.01, 0.08, 0.16, and 0.32 mg/L DA-6.

As shown in Figures 1(d) and 5, DA-6 stimulated the regeneration of hexaploid leaf and petiole explants at optimal concentrations of 0.08 and 0.16 mg/L, respectively. In contrast, supplementation with DA-6 did not enhance the regeneration of root explants, and 0.08 mg/L or higher DA-6 dramatically inhibited regeneration. Different from the explants of diploid, triploid, and tetraploid plants tested above, hexaploid explants produced a large number of calluses under higher DA-6 concentrations, with root and petiole explants producing more callus than that produced by leaf explants (Figure 5).

### 3.2. Effects of DA-6 on the Growth of Buds with Different Ploidy Levels

Buds isolated from diploid, triploid, tetraploid, and hexaploid explant tissues were inoculated in medium supplemented with various concentrations of DA-6, and the effects of DA-6 on the growth of buds were evaluated (see Figure 9, Table 4).

**Figure 6:**
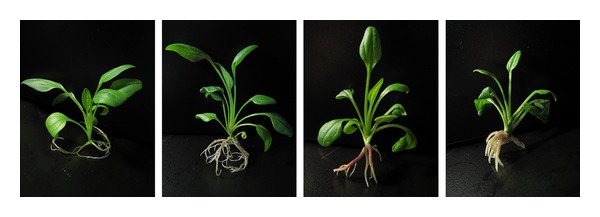
Comparison of the effects of DA-6 on the growth of diploid buds. Photographs from left to right: 0, 0.08, 0.16, and 0.32 mg/L DA-6.

As shown in Table 1 and Figure 6, DA-6 effectively enhanced the growth of diploid buds. Among all the concentrations tested, 0.08 mg/L was the most suitable for improving plant height and weight, the root weight and number, length of primary root, and total root length; the higher concentration of 0.16 mg/L was the most suitable for improving root thickness and the root/top ratio. All of the growth parameters listed in Table 1 were significantly improved (*P* < 0.05).

**Figure 7:**
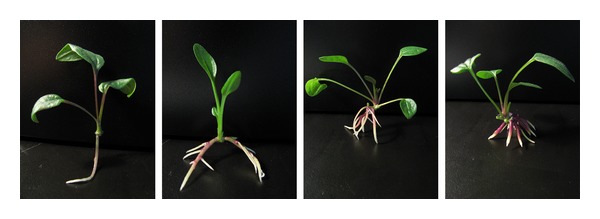
Comparison of the effects of DA-6 on the growth of triploid buds. Photographs from left to right: 0, 0.08, 0.16, and 0.32 mg/L.

DA-6 also effectively enhanced the growth of triploid buds (Table 2 and Figure 7). Among all the concentrations tested, 0.08 mg/L was the most suitable for improving the root/top ratio, while 0.16 mg/L was optimal for improving plant height and weight, root weight and thickness, and total root length. The highest concentration used in this study (0.32 mg/L) was as effective as 0.16 mg/L for improving plant weight and root thickness. Notably, supplying DA-6 to the medium decreased the length of the primary root of the bud in a concentration-dependent manner.

**Figure 8:**
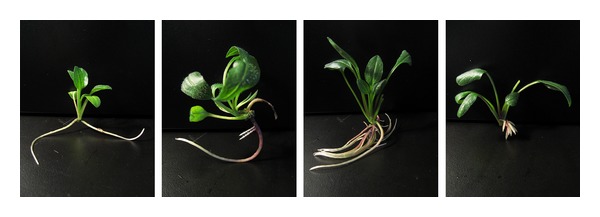
Comparison of the effects of DA-6 on the growth of tetraploid buds. Photographs from left to right: 0, 0.08, 0.16, and 0.32 mg/L DA-6.

**Figure 9:**
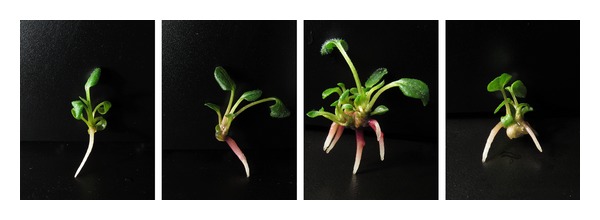
Comparison of the effects of DA-6 on the growth of hexaploid buds. Photographs from left to right: 0, 0.08, 0.16, and 0.32 mg/L DA-6.

As shown in Table 3 and Figure 8, DA-6 effectively enhanced the growth of tetraploid buds. Among all the concentrations tested, 0.08 mg/L was the most suitable for improving the length of the primary root and the root/top ratio, while 0.16 mg/L DA-6 was required to optimal improvement of plant height and weight, root weight, total root number, and total root length. The highest concentration of DA-6 used in this study (0.32 mg/L) was most suitable for improvement of root thickness. However, when used at a concentration of 0.32 mg/L, DA-6 decreased the length of the primary root.

Application of DA-6 at a concentration of 0.08 mg/L improved root weight, length of the primary root, and the root/top ratio in hexaploid plants, while DA-6 at both 0.08 and 0.16 mg/L improved root thickness. Plant height and weight, total root number, and total root length were not significantly affected by the application of DA-6 at the range of concentrations tested.

## 4. **Discussion and Conclusion**

As has been well documented in almost all university textbooks on plant physiology, low working concentrations and large differences in effective concentrations in different organs are 2 of the most important features of plant growth regulators (plant hormones). The results of the present experiments confirmed that DA-6 may be a novel plant growth regulator. However, the fact that explants of higher ploidy levels were more sensitive to the addition of DA-6 in the medium was contradictory to our expectations because we previously demonstrated that explants with higher ploidy levels require higher concentrations of BA to induce adventitious bud formation [21] and to stimulate axillary bud proliferation [20]. Additionally, shoots with higher ploidy levels require higher concentrations of NAA for the initiation of rooting [11]. Therefore, despite the insights provided by our present study, it is still unclear why tissues with higher ploidy levels were more sensitive to DA-6.

BA and NAA are plant growth regulators commonly used in tissue cultures for the induction of adventitious buds formation. BA is a cytokinin that stimulates cell division and serves as a key factor for triggering bud regeneration. In purple coneflower plants, BA has also been adopted for the efficient induction of adventitious bud formation. For example, Choffe et al. used 2.5 *μ*M BA alone without NAA and obtained an average of 8.1 regenerants (shoots and somatic embryos) per petiole explant [7], while Koroch et al. used 4.44 *μ*M BA with 0.054 *μ*M NAA and obtained 7.7 shoots per leaf explant [8]. In the present experiments, we did not obtain a high regeneration frequency comparable with those reported by Choffe et al. and Koroch et al.; however, in a previous work, we found significant variations in adventitious bud regeneration abilities among different genotypes; additionally, altering the BA concentration had only limited effects on the regeneration efficiencies in genotypes with low regeneration abilities [10]. The results of the present experiments suggested that supplementing the culture medium with a certain concentration of DA-6 was able to further enhance the stimulating effects of BA on bud regeneration.

Growth of the regenerated buds to intact purple coneflower plants did not seem to be difficult because rooting of the buds could be achieved simply by inoculating the isolated buds into basal medium [7–9] or to a medium supplemented with an auxin (1.5 mg/L IBA) [22]. However, the present experiments clearly showed that without the application of DA-6, the quality of the rooted plantlets was poor in many parameters, such as plant height, plant weight, root weight, and root number. By adding a certain concentration of DA-6 to the medium, the growth of the isolated buds was effectively improved for plants with all ploidy levels examined.

No studies have demonstrated that DA-6 may inhibit plant growth. Moreover, in a recent report on the dissipation of DA-6 applied to pak choi (Chinese cabbage), cotton, and soil, researchers found that the dissipation half-life of DA-6 was relatively short (5.4–8.2, 1.1–2.2, and 1.5–1.9 days in cotton crop, pak choi, and soil, resp.) [17]. As a consequence of our study, some of the plantlets recovered from DA-6-treated medium were transplanted into the field, where they grew normally to maturity, with the exception of hexaploid plants. High seed setting rates were observed for diploid plants, while much lower seed setting rates were observed for tetraploid and triploid plants. Hexaploid plants, regardless of whether they were treated with DA-6, continued to grow slowly and did not develop flowers, despite being transplanted into the field at the same time as diploid, triploid, and tetraploid plants. Therefore, from the above observation, we concluded that DA-6 had little influence on the fertility of purple coneflower plants, especially for diploid plants.

Polyploid plants [23, 24], including species such as purple coneflower [25], have improved abilities to produce secondary metabolites. Because of this, creation of polyploid plants is one of the strategies for breeding new varieties with higher cultivation value. However, polyploid plants also have many disadvantages [26], and sterility is one of the disadvantages that occurs most frequently. As triploid and autopolyploid plants, such as tetraploid and hexaploid plants, have theoretically low fertility, establishment of efficient *in vitro* propagation culture methods is indispensible for practical cultivation of purple coneflower plants with these ploidy levels in the field.

Based on the present experimental results, we conclude that, when used at suitable concentrations, DA-6 can be used as a plant growth regulator in *in vitro* cultures of purple coneflower plants with various ploidy levels (Table 5). Application of the concentrations listed in the table permitted us to obtain the greatest number and highest quality of regenerated plantlets.

In conclusion, the results of the present experiments demonstrated the positive effects of DA-6 on adventitious buds formation and bud growth in purple coneflower plants and suggested that this plant growth regulator should be further tested in tissue cultures of additional plant species.

## Figures and Tables

**Table 1:** Effects of different concentrations of DA-6 on the growth of diploid buds*.

Concentration of DA-6 (mg/L)	Plant height (mm)	Weight per plant (mg)	Root weight per plant (mg)	Diameter of thickest root (*μ*m)	Total root no.	Length of primary root (mm)	Total root length (mm)	Root/top ratio
0	45.44 ± 3.66^b∗∗^	291.89 ± 13.39^b^	28.89 ± 1.41^c^	965.93 ± 36.69^c^	5.89 ± 0.65^b^	41.88 ± 3.52^a^	78.44 ± 8.99^c^	11.72 ± 0.78^c^
0.08	63.11 ± 3.47^a^	679.11 ± 31.89^a^	190.33 ± 8.71^a^	1421.06 ± 109.45^b^	26.78 ± 2.59^a^	44.77 ± 3.72^a^	217.78 ± 26.34^a^	47.73 ± 3.73^a^
0.16	48.22 ± 3.23^b^	344.89 ± 25.82^b^	96.56 ± 8.17^b^	1739.05 ± 45.42^a^	9.00 ± 0.47^b^	25.88 ± 1.18^b^	133.22 ± 10.61^b^	40.88 ± 5.38^b^
0.32	30.11 ± 2.60^c^	221.89 ± 21.00^c^	59.89 ± 5.25^d^	1318.68 ± 80.53^b^	8.78 ± 0.81^b^	14.77 ± 1.41^c^	114.89 ± 17.46^bc^	37.35 ± 7.15^b^

*Values for plant height, weight per plant, root weight per plant, diameter of thickest root, total root no., length of primary root, total root length, and root/top ratio are expressed as the mean ± SD.

**Data in the same column followed by different letters are significantly different by Duncan's test at *P* < 0.05.

**Table 2:** Effects of different concentrations of DA-6 on the growth of triploid buds*.

Concentration of DA-6 (mg/L)	Plant height (mm)	Weight per plant (mg)	Root weight per plant (mg)	Diameter of thickest root (*μ*m)	Total root no.	Length of primary root (mm)	Total root length (mm)	Root/top ratio
0	35.40 ± 4.52^a∗∗^	174.40 ± 24.65^b^	27.20 ± 4.38^c^	1310.42 ± 88.11^b^	1.10 ± 0.18^b^	32.75 ± 2.72^a^	35.50 ± 5.18^b^	22.21 ± 3.43^c^
0.08	36.10 ± 3.26^a^	186.10 ± 19.78^b^	76.10 ± 12.14^b^	1719.01 ± 54.83^a^	7.30 ± 1.14^a^	29.10 ± 3.53^a^	49.60 ± 8.27^b^	78.58 ± 10.79^a^
0.16	45.10 ± 4.70^a^	466.80 ± 19.81^a^	160.70 ± 16.10^a^	1818.27 ± 12.27^a^	8.00 ± 1.01^a^	21.20 ± 2.30^b^	81.20 ± 15.26^a^	57.24 ± 7.36^b^
0.32	36.20 ± 5.45^a^	478.60 ± 14.67^a^	84.10 ± 4.44^b^	1820.79 ± 18.77^a^	9.70 ± 0.73^a^	6.20 ± 0.55^c^	40.60 ± 5.67^b^	33.61 ± 1.74^c^

*Values for plant height, weight per plant, root weight per plant, diameter of thickest root, total root no., length of primary root, total root length, and root/top ratio are expressed as the mean ± SD.

**Data in the same column followed by different letters are significantly different by Duncan's test at *P* < 0.05.

**Table 3:** Effects of different concentrations of DA-6 on the growth of tetraploid buds*.

Concentration of DA-6 (mg/L)	Plant height (mm)	Weight per plant (mg)	Root weight per plant (mg)	Diameter of thickest root (*μ*m)	Total root no.	Length of primary root (mm)	Total root length (mm)	Root/top ratio
0	32.50 ± 2.40^b∗∗^	247.80 ± 17.76^c^	50.20 ± 4.84^b^	1215.99 ± 48.50^d^	2.30 ± 0.45^c^	41.40 ± 4.22^b^	86.10 ± 15.67^bc^	29.98 ± 3.36^b^
0.08	34.70 ± 3.65^ab^	453.30 ± 49.96^b^	103.90 ± 23.77^b^	1858.95 ± 29.32^c^	4.30 ± 0.42^c^	61.80 ± 6.58^a^	115.10 ± 12.36^b^	31.10 ± 5.05^b^
0.16	46.10 ± 5.41^a^	699.10 ± 86.03^a^	317.60 ± 59.31^a^	2081.84 ± 32.95^b^	16.50 ± 1.48^a^	54.90 ± 9.08^ab^	170.00 ± 26.90^a^	82.00 ± 12.76^a^
0.32	36.40 ± 4.46^ab^	339.80 ± 73.63^bc^	68.50 ± 14.07^b^	2243.96 ± 38.58^a^	11.40 ± 1.00^b^	12.10 ± 1.37^c^	43.30 ± 7.22^c^	35.36 ± 5.30^b^

*Values of plant height, weight per plant, root weight per plant, diameter of thickest root, total root no., length of primary root, total root length, and root/top ratio are expressed as the mean ± SD.

**Data in the same column followed by different letters are significantly different by Duncan's test at *P* < 0.05.

**Table 4:** Effects of different concentrations of DA-6 on the growth of hexaploid buds*.

Concentration of DA-6 (mg/L)	Plant height (mm)	Weight per plant (mg)	Root weight per plant (mg)	Diameter of thickest root (*μ*m)	Total root no.	Length of primary root (mm)	Total root length (mm)	Root/top ratio
0	15.60 ± 1.71^ab∗∗^	212.70 ± 17.38^ab^	63.20 ± 12.47^b^	1420.00 ± 62.91^d^	2.30 ± 0.37^ab^	27.88 ± 3.02^bc^	30.30 ± 4.67^ab^	42.93 ± 8.13^b^
0.08	20.40 ± 3.28^a^	279.40 ± 42.81^a^	116.60 ± 65.02^a^	1650.09 ± 98.80^c^	3.70 ± 1.09^a^	44.77 ± 5.12^a^	56.00 ± 11.65^a^	75.15 ± 10.26^a^
0.16	14.10 ± 2.69^ab^	170.70 ± 30.17^b^	53.40 ± 15.43^b^	2495.55 ± 81.22^a^	2.20 ± 1.02^ab^	36.25 ± 8.50^ab^	36.70 ± 12.13^ab^	54.52 ± 10.70^ab^
0.32	11.20 ± 1.91^b^	143.90 ± 25.62^b^	39.40 ± 11.01^b^	2180.24 ± 66.53^b^	0.90 ± 0.18^b^	17.75 ± 2.54^c^	14.40 ± 3.81^b^	36.18 ± 8.36^b^

*Values of plant height, weight per plant, root weight per plant, diameter of thickest root, total root no., length of primary root, total root length, and root/top ratio are expressed as the mean ± SD.

**Data in the same column followed by different letters are significantly different by Duncan's test at P < 0.05.

**Table 5:** Summary of suitable concentrations of DA-6 for application in various cultures.

Purpose of culture	Explant source	Ploidy levels	DA-6 concentration (mg/L)
Adventitious bud regeneration	Leaf	Diploid	0.16
		Triploid	0.16
		Tetraploid	0.08
		Hexaploid	0.08
	Petiole	Diploid	0.08
		Triploid	0.01
		Tetraploid	0.01
		Hexaploid	0.16
	Root	Diploid	0.01
		Triploid	0.01
		Tetraploid	0.01
		Hexaploid	0
Growth of regenerated buds		Diploid	0.08
		Triploid	0.16
		Tetraploid	0.16
		Hexaploid	0.08

## Abbreviations

BA: 6-Benzyladenine
DA-6: Diethyl aminoethyl hexanoate
IBA: Indole-butyric acid
MS: Murashige and Skoog(1962)
NAA: Naphthaleneacetic acid.
